# Pericardial Diffuse Large B-Cell Lymphoma Diagnosed Via Computed Tomography–Guided Biopsy

**DOI:** 10.1016/j.jaccas.2025.105070

**Published:** 2025-09-24

**Authors:** Toshiya Yoshida, Midori Nanamatsu, Shunichi Asano, Takuya Narita, Miki Terauchi, Akira Hirasawa, Yukio Kakuta, Kazuhiko Yumoto

**Affiliations:** a Department of Cardiology, Yokohama Rosai Hospital, Yokohama, Japan; b Department of Cardiovascular Surgery, Yokohama Rosai Hospital, Yokohama, Japan; c Department of Radiology, Yokohama Rosai Hospital, Yokohama, Japan; d Department of Hematology, Yokohama Rosai Hospital, Yokohama, Japan; e Department of Diagnostic Pathology, Yokohama Rosai Hospital, Yokohama, Japan

**Keywords:** CT-guided biopsy, diffuse large B-cell lymphoma, pericardial tumor

## Abstract

**Background:**

Primary pericardial lymphomas are rare. We describe a case of pericardial diffuse large B-cell lymphoma (DLBCL) diagnosed via computed tomography (CT)–guided biopsy.

**Case Summary:**

A 57-year-old man presented with chest pain and weight loss for 1 month. Multiple diagnostic modalities revealed a pericardial mass with effusion. Pericardiocentesis was performed, and cytology suggested malignant lymphoma; however, the procedure was complicated by pneumothorax owing to mediastinal distortion from the pectus excavatum and minimal effusion. After multidisciplinary discussion, CT-guided biopsy was performed, which confirmed DLBCL. Polatuzumab vedotin, rituximab/cyclophosphamide, doxorubicin, and prednisone were initiated, resulting in tumor shrinkage.

**Discussion:**

Early histopathological diagnosis is essential for treating malignant lymphoma. In this case, CT-guided biopsy was selected as the safest and most effective approach and was successfully performed without complications.

**Take-Home Messages:**

A multidisciplinary approach is necessary for early diagnosis of pericardial lymphoma. CT-guided biopsy can be effective in certain cases.

## Graphical Abstract

**Figure:**
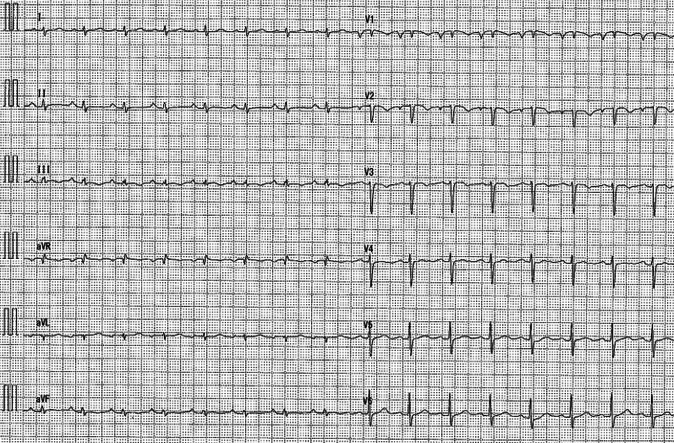

## History of Presentation

A 57-year-old man was referred to our institution because of chest pain aggravated by inspiration and 5-kg weight loss over the past month. At presentation, vital signs were stable. Physical examination revealed a painless, elastic, firm 2-cm mass in the right parotid gland, and a pericardial friction rub was auscultated.

**Take-Home Messages**

• CT-guided biopsy can be useful for diagnosis in certain cases of pericardial malignant lymphoma, especially when pericardiocentesis is challenging owing to anatomical features or other factors.
• Early definitive diagnosis is crucial, and a multidisciplinary approach is essential to determine the optimal diagnostic strategy.

## Past Medical History

The patient had pectus excavatum. There was no history of organ transplantation, autoimmune disease, or malignancy. The patient did not take any medications.

## Differential Diagnosis

Pericarditis was suspected based on chest pain aggravated by inspiration and pericardial friction rub. In addition, the presence of a parotid gland mass raised the possibility of concurrent malignancy and carcinomatous pericarditis as differential diagnoses.

## Investigations

**Figure 1:**
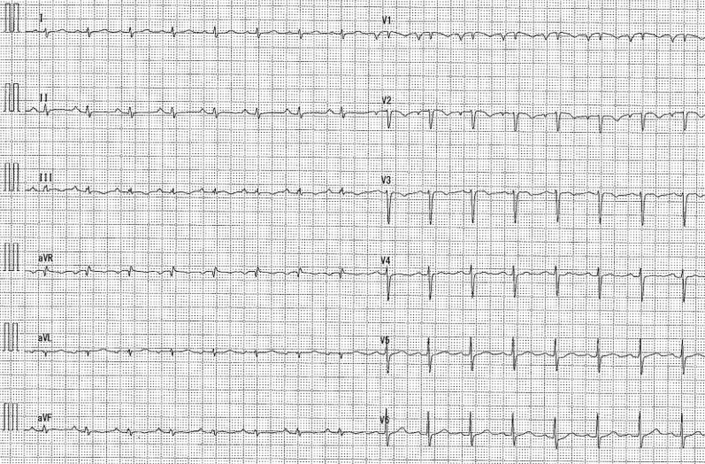
12-Lead Electrocardiogram on Admission A 12-lead electrocardiogram showing sinus rhythm, low voltage in the limb leads, and T-wave inversions in leads V_1_ to V_3_.

The 12-lead electrocardiogram showed sinus rhythm, low voltage in the limb leads, T-wave inversions in leads V_1_ to V_3_, and a clockwise rotation pattern (Figure 1). Blood tests results revealed elevated levels of C-reactive protein (3.87 mg/dL), brain natriuretic peptide (107.6 pg/mL), and soluble interleukin-2 receptor (943 U/L). The patient tested negative for human immunodeficiency virus antibodies.

**Figure 2:**
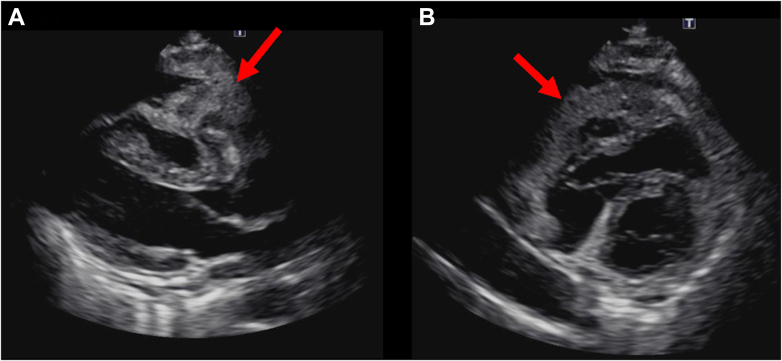
Initial Transthoracic Echocardiography Imaging reveals a mass attached to the right atrium and right ventricle (red arrows) and pericardial effusion in the pericardial cavity. (A) Parasternal long-axis view and (B) parasternal long-axis view.

**Figure 3:**
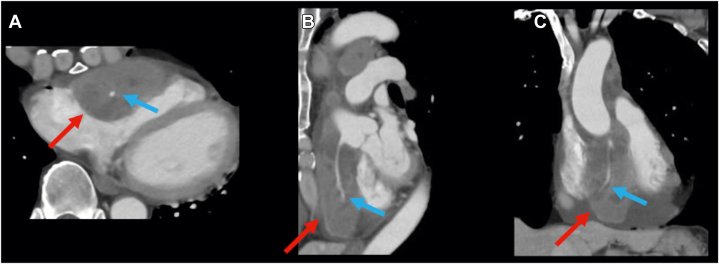
Contrast-Enhanced Computed Tomography Imaging reveals a mass measuring 60 × 35 × 90 mm in the pericardial cavity along the anterior wall of the right atrium and right ventricle, without extension into the cardiac chambers (red arrows) and surrounding the right coronary artery (blue arrows); despite that, blood flow was preserved. (A) Axial view. (B) Sagittal view. (C) Coronal view.

**Figure 4:**
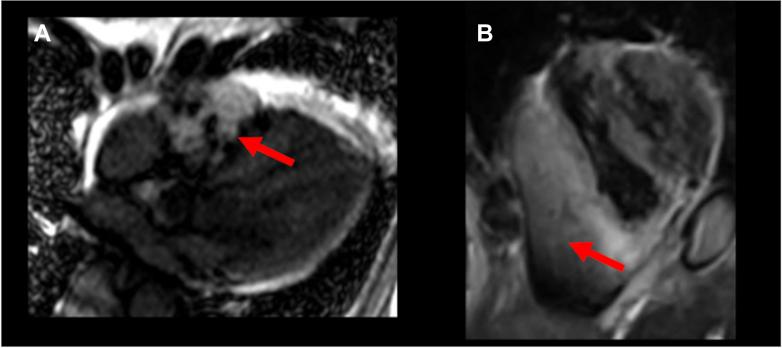
Cardiac Magnetic Resonance The mass (red arrows) shows heterogeneous, indeterminate enhancement on 4-chamber late gadolinium enhancement image (A) and variable intensity on short-axis fat-suppressed T2-weighted image (B).

Transthoracic echocardiography (TTE) showed that the left ventricular ejection fraction was 62%, with no valvular disease. A pericardial mass and an effusion were observed in contact with the right atrium and ventricle, respectively (Figure 2, Video 1). Contrast-enhanced computed tomography (CT) revealed a pericardial mass measuring 60 × 35 × 90 mm along the anterior wall of the right atrium and ventricle. Although the mass surrounded the right coronary artery, blood flow was preserved (Figure 3, Video 2). A right parotid gland mass and multiple enlarged lymph nodes were observed in the right supraclavicular fossa, right axilla, and mediastinum. Cardiac magnetic resonance revealed indeterminate enhancement of the mass on late gadolinium-enhanced images and variable intensity on T2-weighted images, suggesting malignancy (Figure 4, Video 3).^1^ A positron emission tomography scan was considered to identify the primary site. As positron emission tomography was not available at our institution and given the need for early diagnosis, we did not pursue this approach.

Because the cardiac magnetic resonance findings suggested malignancy, a biopsy was considered necessary. Subsequently, pericardiocentesis was performed; however, only 3 mL of fluid sample could be obtained. Although a small pneumothorax developed, the respiratory status of the patient remained unchanged, and the pneumothorax did not worsen. Gradual improvement of the pneumothorax was noted with observation alone. The pericardial fluid was serosanguineous, with significantly elevated lactate dehydrogenase levels (1,502 U/L). Cytologic examination revealed class IV findings with mesothelial cells and large lymphocyte-like atypical cells, suggestive of malignant lymphoma. However, because of the small volume of the pericardial fluid, immunostaining and flow cytometry could not be performed. Malignant lymphoma was suspected; however, a definitive diagnosis required a histopathologic examination. Therefore, we explored the most appropriate biopsy method through discussions among the heart team, radiology, hematology, and pathology departments. Repeat pericardiocentesis was considered an option. However, this was technically challenging because of anatomical features, including the presence of pectus excavatum, which limited the safe puncture space, as the lung overlapped the puncture trajectory. The patient had already developed iatrogenic pneumothorax; therefore, repeat pericardiocentesis could cause recurrence or worsening of the pneumothorax. Furthermore, a small effusion volume indicated a low probability of obtaining a sufficient sample size. Therefore, repeat pericardiocentesis was deemed unfeasible.

**Figure 5:**
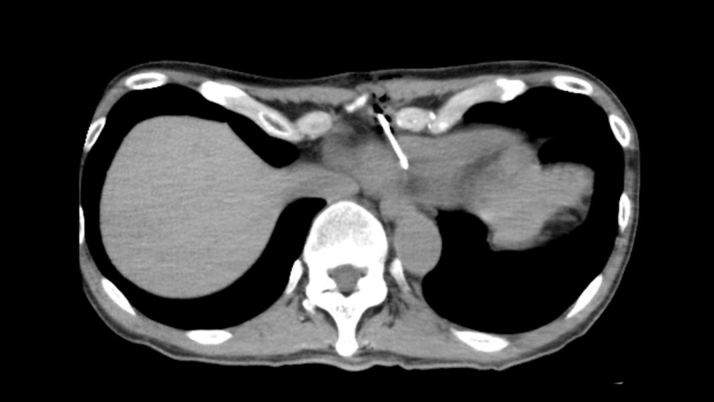
Computed Tomography–Guided Biopsy of Pericardial Tumor Under local anesthesia, 3 samples were obtained using an 18-gauge biopsy needle via the left lower parasternal approach. The procedure was completed without any complications.

**Figure 6:**
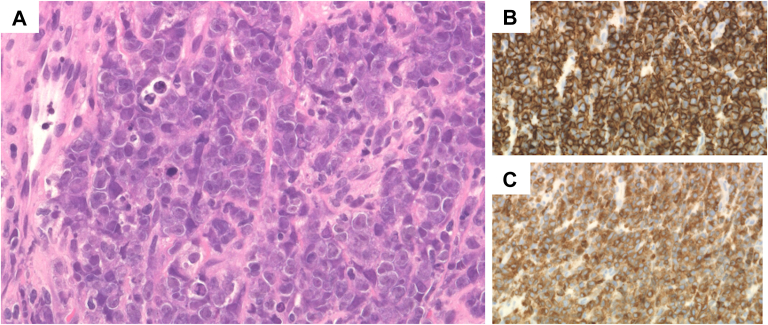
Histologic and Immunohistochemical Assessment of Computed Tomography–Guided Biopsy Round cell tumor in the pericardium. (A) Hematoxylin and eosin staining. (B) High CD20 expression and (C) high CD79a expression (original magnification, 40×).

We determined that CT-guided biopsy could be performed safely by avoiding critical structures, including the right coronary artery, and minimizing the risk of pneumothorax worsening. The procedure was performed under local anesthesia. CT was used to identify the shortest and safest trajectory that avoided the coronary arteries, thoracic organs, and lung tissue, and a biopsy was performed using an 18-gauge needle via the left lower parasternal approach, corresponding to the area along the left edge of the fifth intercostal space (Figure 5). Three passes were performed, which resulted in adequate tissue sampling. TTE and chest radiography revealed no signs of tamponade or pneumothorax, with the patient remaining stable. Histologic examination revealed a round cell tumor in the pericardium. Immunohistochemical staining showed the following results: AE1/AE3 (−), LCA (+), CD3 (−), CD20 (+), CD7a (+), CD10 (+), Bcl-6 (−), MUM-1 (+), CD30 (+), and INSM1 (−), leading to a definitive diagnosis of diffuse large B-cell lymphoma (DLBCL), germinal center B-cell subtype (Figure 6).

## Management

**Figure 7:**
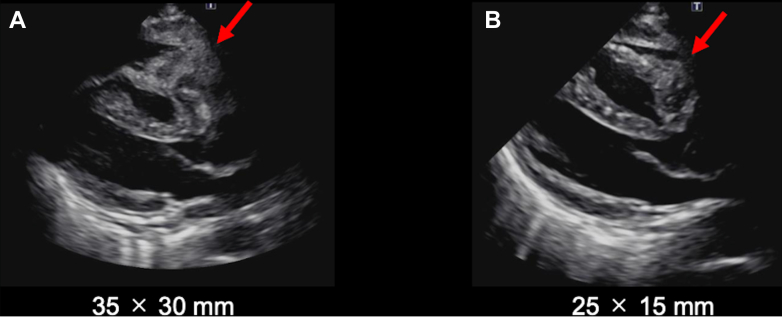
Changes in Tumor Size on Transthoracic Echocardiography The pericardial tumor (red arrows), which measured 35 × 30 mm at admission (A), decreased in size to 25 × 15 mm at discharge (B) after 2 cycles of chemotherapy.

On day 15 of hospitalization, treatment with polatuzumab vedotin, rituximab/cyclophosphamide, doxorubicin, and prednisone therapy was initiated. After 2 cycles, TTE showed tumor reduction (Figure 7). The patient was discharged home on the 44th day of hospitalization.

## Follow-Up

The patient is currently undergoing chemotherapy and attending follow-up at the cardiology and hematology departments of our hospital.

## Discussion

Primary cardiac lymphomas are uncommon malignancies that primarily manifest as aggressive B-cell lymphoma. This is mainly observed in immunocompetent patients, accounting for 1.3% and 0.5% of extranodal lymphomas. Cardiac metastasis from systemic lymphoma is more common than primary cardiac lymphoma.^1^, ^2^, ^3^ Distinguishing between primary and secondary cardiac lymphomas is inherently challenging, particularly in cases with multiple organ involvement, as in the present case. Based on the World Health Organization classification^4^ and National Cancer Institute statements^5^ regarding primary site determination in lymphoma, we considered the pericardial lesion to be the largest primary lesion despite the occurrence of multiple enlarged lymph nodes. Primary cardiac lymphomas usually arise in the right atrium and ventricle and are contiguous with the pericardium.^1^^,^^2^ However, primary pericardial lymphomas are rare, with only a few reported cases. In the present case, the primary location of the tumor was considered to be the pericardial cavity; notably, none of the imaging modalities demonstrated definitive evidence of intracardiac extension, supporting the diagnosis of primary pericardial lymphoma.

We considered 3 options for pericardial lesion biopsy: 1) repeat pericardiocentesis; 2) surgical biopsy; and 3) CT-guided biopsy. Repeat pericardiocentesis was challenging because of anatomical features, and it was thought that it would not yield a sufficient sample. Regarding surgical biopsy, simultaneous maximal tumor resection was considered because repeat thoracotomy was unfavorable owing to its invasiveness. Intraoperative manipulation near cardiac structures may result in valvular dysfunction or conduction disturbances, whereas surgical resection does not improve the prognosis if the diagnosis is lymphoma.^6^ Therefore, surgery was reserved as a salvage option in cases of cardiac tamponade or hemodynamic collapse, but was not pursued under stable conditions. Therefore, we determined that obtaining a definitive diagnosis via CT-guided biopsy and initiating chemotherapy promptly would be the best strategy.

Biopsy was performed without complications, allowing rapid diagnosis and early initiation of treatment. A recent study reported a DLBCL case with multiorgan involvement, including pericardial lesions, in which CT-guided biopsy of extracardiac lesions led to a definitive diagnosis.^7^ Another study investigating CT-guided biopsy in 12 cases of cardio-pericardial masses reported that a definitive diagnosis was achieved in all cases. Although minor complications, such as small hemothorax and mediastinal emphysema, were observed in 1 case each, both were mild. These findings suggested that CT-guided biopsy may be a useful approach for diagnosing cardio-pericardial masses.^8^ In the present case, the admission 12-lead electrocardiogram showed T-wave inversions in leads V_1_ to V_3_ and a clockwise rotation pattern. These findings may reflect not only the thoracic deformity associated with pectus excavatum, but also right heart strain and anatomical displacement caused by compression from the pericardial tumor. CT-guided biopsy may be a useful diagnostic tool even in patients with anatomical abnormalities, as in this case. In the present case, a CT-guided biopsy was performed directly on a presumed primary pericardial tumor, leading to the diagnosis of DLBCL. Although this approach may be a valuable option in similar cases, a disadvantage of CT-guided biopsy is the increased exposure of both the operator and the patient to radiation.^9^ Careful consideration of the tumor location, patient background, radiation exposure, and individual case factors is essential.

Until recently, rituximab, cyclophosphamide, doxorubicin, vincristine, and prednisone therapy was the standard treatment for DLBCL. However, in 2022, the efficacy of polatuzumab vedotin, an antibody-drug conjugate targeting CD79b, in combination with *rituximab, cyclophosphamide, doxorubicin, and prednisone* was reported,^10^ making it a viable treatment option. In the present case, *polatuzumab, rituximab/cyclophosphamide, doxorubicin, and prednisone* therapy was administered for treating a primary pericardial malignant lymphoma. Although the tumor showed a short-term reduction in size and the patient progressed favorably, careful long-term follow-up is warranted.

## Conclusions

Pericardial malignant lymphoma is a rare disease; therefore, there are no established methods for biopsy, and early diagnosis and chemotherapy initiation are crucial. The present case suggested that CT-guided biopsy is a useful option for diagnosing primary pericardial malignant lymphoma.

## Funding Support and Author Disclosures

The authors have reported that they have no relationships relevant to the contents of this paper to disclose.

## Appendix

Video 1 Initial Transthoracic Echocardiographic Examination In the apical right ventricular inflow long-axis view, the tumor appears heterogeneously echogenic and is visualized adjacent to the right atrium and ventricle; however, no apparent extension into the cardiac chambers is observed.

Video 2 Contrast-Enhanced Computed Tomography The axial view reveals a mass measuring 60 × 35 × 90 mm in the pericardial cavity along the anterior wall of the right atrium and right ventricle. Although the tumor externally compresses the right side of the heart, no clear intracardiac extension is observed. In addition, thoracic deformity owing to pectus excavatum is revealed.

Video 3 Cardiac Magnetic Resonance In the 4-chamber perfusion view, the tumor is situated within the pericardial cavity, adjacent to both the right atrium and the right ventricle. The tumor externally compresses the right side of the heart without obvious direct invasion into either chamber.
